# Detection of Plastic Granules and Their Mixtures

**DOI:** 10.3390/s23073441

**Published:** 2023-03-24

**Authors:** Roman-David Kulko, Alexander Pletl, Andreas Hanus, Benedikt Elser

**Affiliations:** 1 Technologie Campus Grafenau, Technische Hochschule Deggendorf, 94481 Grafenau, Germany; 2 Sesotec GmbH, Regener Straße 130, 94513 Schönberg, Germany

**Keywords:** plastic granulate, VIS-NIR spectroscopy, spectral data mixing, machine learning, fusion model, reconstruction of spectra

## Abstract

Chemically pure plastic granulate is used as the starting material in the production of plastic parts. Extrusion machines rely on purity, otherwise resources are lost, and waste is produced. To avoid losses, the machines need to analyze the raw material. Spectroscopy in the visible and near-infrared range and machine learning can be used as analyzers. We present an approach using two spectrometers with a spectral range of 400–1700 nm and a fusion model comprising classification, regression, and validation to detect 25 materials and proportions of their binary mixtures. one dimensional convolutional neural network is used for classification and partial least squares regression for the estimation of proportions. The classification is validated by reconstructing the sample spectrum using the component spectra in linear least squares fitting. To save time and effort, the fusion model is trained on semi-empirical spectral data. The component spectra are acquired empirically and the binary mixture spectra are computed as linear combinations. The fusion model achieves very a high accuracy on visible and near-infrared spectral data. Even in a smaller spectral range from 400–1100 nm, the accuracy is high. The visible and near-infrared spectroscopy and the presented fusion model can be used as a concept for building an analyzer. Inexpensive silicon sensor-based spectrometers can be used.

## 1. Introduction

Plastic granulate (PG) is available in a variety of polymers and colors. It is manufactured by a chemical polymerization process. After poliymerization, the plastic is melted and cut into PG. At this stage, the PG is pure and serves as a raw material for the production of plastic parts. These machines require pure PG as the starting material. To prodce different parts, the machine parameters and the raw material have to be changed. The wrong PG may be used or not all of the previously used PG may be removed from the machine. As a result, the parts produced do not fulfill the requirements and are sorted out. This means a loss of resources for the manufacturer. Ideally, machines should be able to analyze raw materials quickly, automatically, and cost-effectively. The detection of plastic is rare in this field due to the expected purity of the starting materials. The detection of plastics plays a crucial role in the sorting of plastic waste. Applied techniques are also relevant to this article. Therefore, we describe common methods of the detection of plastics from the field of plastic sorting.

Sorting plastics has traditionally relied on a combination of manual labor and physical methods. More recently, solutions have focused on automated plastics sorting systems that use spectroscopy in combination with machine learning methods. The analysis of spectral data using machine learning methods has been known in the field of chemometrics for decades.

Chemometrics has been applied extensively for quality control in the food [1] and pharmaceutical industries [2], quality assessment and ripeness determination of fruits and vegetables [3], environmental modeling [4], and forensics [5]. Chemometric techniques have only recently gained popularity in the area of plastic waste [6]. Some systems have explored the use of chemometrics, in which chemical data from spectroscopic methods [7] are used to automatically analyze plastics. A comprehensive review of various physical and chemometric methods for municipal solid waste sorting has recently been carried out by Gundupalli et al. [8]. Chemometrics is therefore predestined for use in the detection of PG and its mixtures.

In general, spectroscopy describes the interaction of electromagnetic radiation with matter. Spectrometers are used to measure the phenomenon. In the case of detection of plastics, setups are used to measure the absorbance in visible and near-infrared (VIS-NIR). The operating principle of the method consists of five steps. (1) A light source, usually a halogen lamp, irradiates the sample. Within the sample, absorption of certain wavelengths of radiation by molecules and scattering of light occur. This results in characteristic changes in the reflected versus the irradiated radiation. (2) The reflected radiation is guided into the spectrometer, e.g., via light guides. (3) In the spectrometer, the reflected radiation is split into wavelength ranges using a monochromator and projected onto a light-sensitive electronic sensor. (4) The electronic sensor generates a signal proportional to the radiation intensity. (5) Finally, computers are used to calculate the absorbance.

Spectroscopy is the analytical tool of choice due to its specificity and speed. Spectroscopy in the VIS-NIR range detects the absorption spectra of the material under investigation. Since each molecule absorbs a specific range of wavelengths in a unique way, this technique can be used to provide a “material fingerprint”.

Near-infrared (NIR) spectroscopy is used in industry and academia. Although spectroscopy applications are fast, single spectrometer setups have the disadvantage of small observation areas and slow information utilization [9]. Single spectrometer setups are also used in portable devices for the manual identification of plastics (cf. Kumagai et al. [10] and Yang et al. [11]).

Analyzers based on NIR hyperspectral imaging are used in industrial recycling processes [12,13,14]. These analyzers work on the same principle as a simple spectrometer, but cover a large observation range with high spatial resolution and use multiple spectrometers simultaneously. In combination with a conveyor belt, these analyzers are capable of analyzing a high material throughput of over 1t/h.

NIR hyperspectral imaging is also used in science [15,16]. Spectral data are preprocessed and analyzed using statistical, machine learning, and deep learning methods [17].

In most cases, it is sufficient to classify the material based on NIR spectra and a classification model. Therefore, various classification algorithms are applied. Kaihara et al. [18] reported the use of NIR spectroscopy and classification and regression trees to discriminate between spectra and types of polymers. Yang et al. [11] reported the use of a principal component analysis, support vector machine, K-nearest neighbor and back propagation neural networks on NIR spectral data. Wu et al. [19] demonstrated the use of a partial least squares regression discriminant analysis. Most of the reported regression models are related to the determination of component concentrations of plastic blends, e.g., low density polyethylene/high density polyethylene [20,21].

Our data pipeline is constructed as a fusion of preprocessing and multiple estimators, and has a process for evaluating the results. In addition, we address a general issue in data-driven examinations, which is to provide a sufficiently large amount of data. This is particularly important when applying deep learning.

In our study, we investigate the mixtures of 25 plastic hlgranulates.

We use both a visible (VIS) and a NIR spectrometer to collect data over a wide range of wavelengths. NIR spectroscopy is our primary source of information to distinguish plastics’ granulate mixtures based on their polymers.

VIS spectroscopy can only help distinguish plastic granulates if they are colored. A spectrum of an observation area is the superposition of reflected and collected light from that area. To distinguish the different mixtures of PG, we apply a machine learning and deep learning approach, respectively. Therefore, appropriate data are needed to fit the model.

In addition to a sufficient number of samples, it is important that the data set contains the necessary amount of variation to cope with in practice. Thus, we implement a spectrum simulator to satisfy these requirements, using semi-empirical mix spectra of 300 combinations. The fusion of pre-processing and application of artificial intelligence allows the accurate detection of materials, binary mixtures, and their compositions in the observation area.

The goal of this paper is to establish a machine-learning-based approach to classify different PG and determine the proportion of each component in granule mixtures. This paper adresses several research gaps and presents a novel method for recognizing materials, their binary mixtures, and the mixing ratio based on VIS and NIR spectral data.

The rest of this paper is structured as follows: Section 2, describes the data and measurement setup in detail. Furthermore, the spectrometers used are presented and the data processing is shown. Section 2.5 introduces the applied deep and machine learning tools for analysis. The design of the fusion model is also demonstrated. Afterwards, the obtained results are reported in Section 3. The paper ends with a brief discussion and conclusion (Section 4 and Section 5).

## 2. Materials and Methods

This section is dedicated to experimental setup, data processing, and modeling techniques.

### 2.1. Spectrometers and Experimental Setup

The experimental setup contains a VIS and a NIR spectrometer. Both spectrometers were purchased from Dr. Licht GmbH [22].

**Figure 1:**
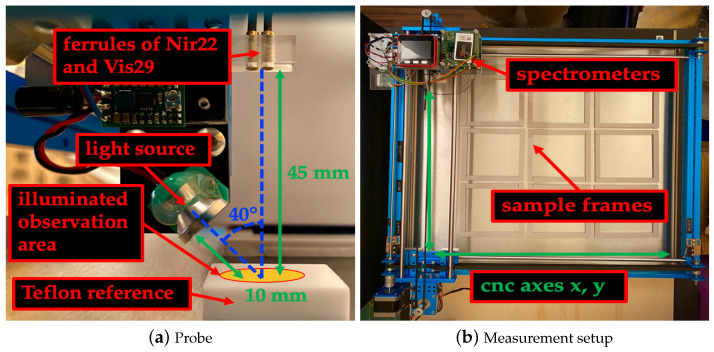
On the left (**a**), the probe consisting of ferrules and lamp is presented. The teflon reference block is also shown. On the right (**b**), the top view of the experimental setup is illustrated.

The Vis29 uses a silicon line sensor with 512 pixels and a grating monochromator. The measuring range is from 148 to 1161 nm. The spectral resolution is less than 10 nm full width at half maximum (FWHM). On the sensor, 2 nm per pixel are imaged. The signal-to-noise ratio (SNR) is greater than 5000. The Nir22 uses an InGaAs line sensor with 128 pixels and a grating monochromator. The measurement range is from 877 to 1902 nm. The spectral resolution is less than 16 nm FWHM. A total of 8.2 nm per pixel are imaged on the sensor. The SNR is greater than 5000. Nir22 is not temperature controlled and there is no temperature compensation. Each spectrometer is equipped with a glass fiber and a ferrule. Both ferrules were held together by a custom-made probe, as shown in Figure 1a.

The focused light source is a lamp consisting of a 0.9 W bulb combined with a reflector. The lamp is located on the same probe as the ferrules. The probe allows the angle and the distance of the lamp and ferrules to the sample to be adjusted. The angle between the lamp and the optical axes of the ferrules was 40°. The angle of the optical axes to the sample is 90°. The distance between the ferrules and the sample was 45 mm. This distance gives an observation area with an diameter of 10 mm for each ferrule. The distance from the lamp to the sample is 10 mm and achieves a maximum illumination of the overlapping observation areas.

To automate the measurements, we modified a two-axes CNC machine, and equipped it with nine custom-made sample frames, each measuring 100 × 100 × 6 mm and mounted with spectrometers and a lamp (Figure 1b). A M5Stack (ESP32) controls the spectrometers, lamp and, cnc machine with custom-made Micropython version 1.14 firmware and control software. With this setup, an area of 300 × 300 mm could be scanned automatically.

A 30 × 30 × 10 mm teflon block served as the spectral reference material. With this setup, the reflectance of samples can be measured.

### 2.2. Plastics

**Figure 2:**
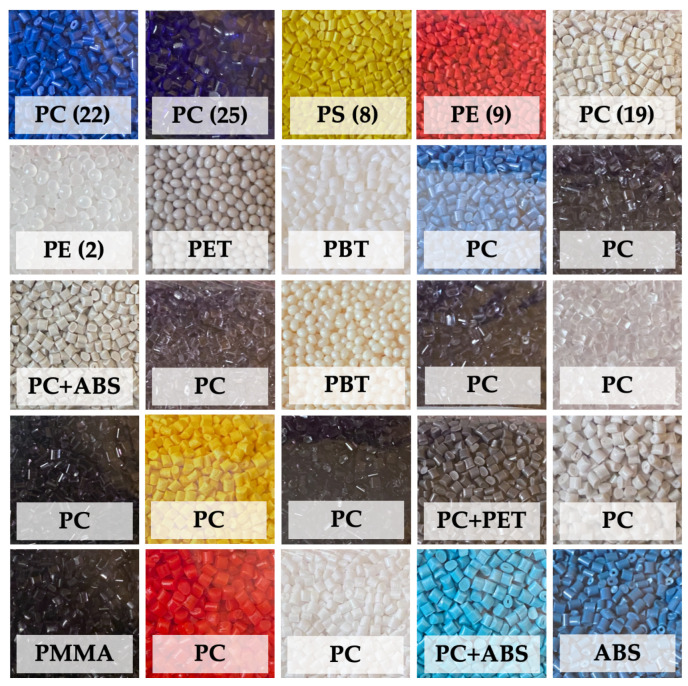
The 25 different types of plastic granulate being studied. Samples mentioned in the article are marked with a number.

We used 25 different PGs as samples. Figure 2 shows sample PGs. The samples mentioned in the article are marked with a number. The samples consist of polymers of polyethylene (PE), polymethylmethacrylate (PMMA), polyethylene terephthalate (PET), polycarbonate (PC), polystyrene (PS), polybutylene terephthalate (PBT), acrylonitrile butadiene styrene (ABS), and co-polymers of PC and PET, and PC and ABS.

The samples varied in color (white, light gray, gray, dark gray, black, dark blue, blue, light blue, yellow, beige, orange, or red), transparency (opaque or transparent) and shape (ellipsoidal or cylindrical). However, all plastic granules had a diameter of approximately 2 to 3 mm.

The different material properties have an influence on the spectroscopic measurements and on the models to be built. The influences are briefly discussed in the following Section 2.3.

### 2.3. Measurement and Data Acquisition

**Figure 3:**
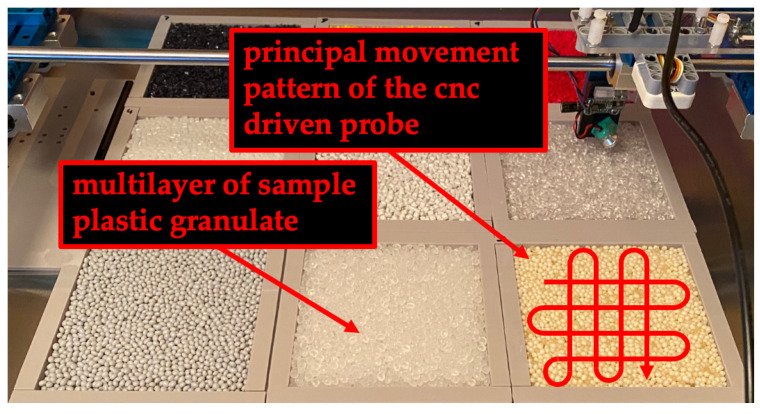
Nine sample frames filled with different plastic granules and probe.

To obtain a spectral data set that includes scan-based effects such as moving total reflections, shadows, and slight variations, we performed measurement distances between the probe and sample surfaces using the modified CNC machine. For each measurement, sample frames were filled with different PG (cf. Figure 3). Each sample frame is scanned to the same pattern with a sampling rate of 20 mm/s. The pattern consists of rows and columns with curved direction changes to the next row or column. This pattern was used to scan the entire 100 × 100 mm sample area in a continuous motion.

During the movement, 50 sample spectra were recorded at room temperature. Each sample spectrum is an average of 10 spectra. Before each scan, teflon was measured for calibration. The calibration measurement is necessary because the instrument needs information as a spectral reference: These reference spectra are recorded with (a) lamp off, $I_{dark}$, and (b) with lamp on, $I_{teflon}$. They are used to transform any other sample intensity, $I_{sample}$, to reflectance using the Equation (1):

(1) $$reflectance=\frac{I_{sample}-I_{dark}}{I_{teflon}-I_{dark}}$$

Reflectance can be converted to absorbance using Equation (2):

(2) $$absorbance=log\left(\frac{1}{reflectance}\right)$$

We applied an algorithm to optimize the dynamic integration time to optain optimal reflectance spectra. Classically, a measurement is performed on a sample using the spectrometer settings specified for the reference, e.g., teflon. However, the intensity of the spectral data can be very low because the diffuse reflectance of a sample can be much lower than that of the reference sample. To ensure high intensity and at the same time optimal SNR for each measurement, it is advisable to adjust the integration time and increase the number of averages. To find the optimal integration time, $it_{opt}$, we proceed as follows:

(1) Measure an intensity spectrum at a typical integration time. (2) Calculate the reflectance. (3) Find the maximum value of the reflectance. (4) $it_{opt}$ is obtained by dividing the typical integration time by the maximum of reflectance. (5) Optional: Correct $it_{opt}$ by multiplying $it_{opt}$ by an empirical factor of 0.95 to ensure that the reflectance is less than 1. (6) Check if $it_{opt}$ is below a threshold of 5 s, in order to avoid too long measurement times or sensor saturation. (7) Finally, repeat the measurement with $it_{opt}$.

**Figure 4:**
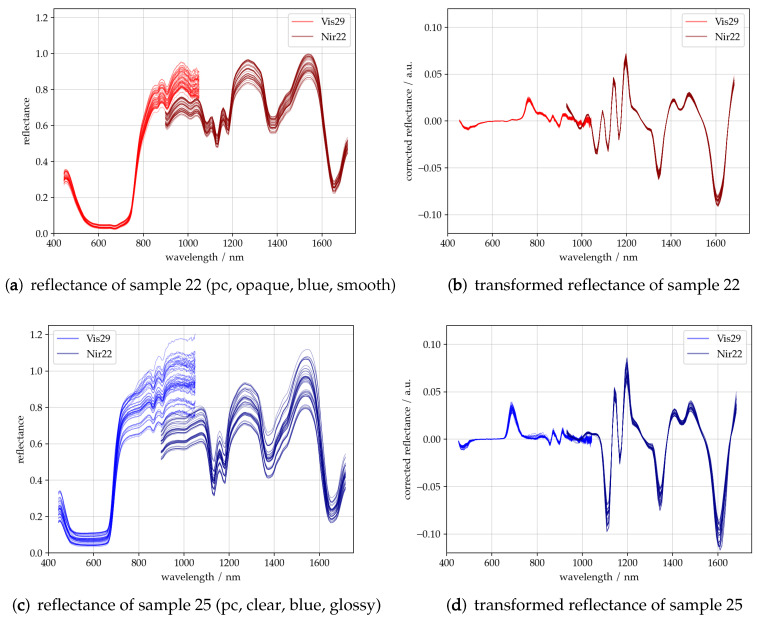
Reflectance spectra recorded by Vis29 and Nir22 of sample 22 (PC, opaque, blue, and smooth), (**a**), and 25 (PC, transparent, blue, and glossy), (**c**). Spectral ranges of the two spectrometers overlap from 877–1050 nm. Savitzky–Golay filter (1st derivative) transformed reflectance spectra of samples 22 (**b**) and 25 (**d**).

Figure 4a,c show typical sets of 50 reflectance spectra recorded by Vis29 and Nir22. The wavelength range of Vis29 covers VIS, which ranges from 400–780 nm, and a small range of about 780–1050 nm of NIR. Compared to the spectrum of Nir22, which covers a wavelength range from 877–1700 nm, similar reflectance bands occur in this overlapping range. The difference between the two reflectance spectra results from the measurement setup. Since no multiplexer was used for the glass fibers and the spectrometer settings were different, the spectrometers were measured at different locations on the same sample.

In general, low reflectance bands in the VIS spectrum can be assigned to electronic transitions, e.g., of dyes in the sample (cf. Figure 4a,c). Bands in the NIR spectrum are assigned to vibrational energy transitions of molecules.

It is important to note that in addition to the absorption of radiation by molecules, the two spectrometers also observe other material properties and effects of the experimental setup. This can be observed in the variance of the spectra and will be briefly discussed. The variation of the baseline is caused by slightly different integration times, varying distances between the sample and the moving measuring head and scattering behavior. Both samples consist of PC. Their color is somewhat similar, as they both possess a shade of blue, but they greatly differ in terms of their transparency and surface characteristics. Sample 22, in Figure 2, consists of blue opaque PG, which has a satin finish. In contrast, sample 25 consists of blue, clear PG, which has a smooth glossy surface. In the case of sample 25, therefore, a greater amount of total reflection is to be expected. In addition, the material is transparent. Therefore, it is also possible that radiation penetrates through this PG to the stainless steel sample holder, reflects to the ferrules, and is measured. Due to the random distribution of the PG, and the resulting intensity fluctuations, the variance of the reflectance is expected to be higher than in sample 22.

As already mentioned, this affects the baseline. An established method of the baseline correction, as used later in this paper, is to apply the Savitzky–Golay filter [23]. Figure 4b,d show the 1st derivative of the reflectance spectra of samples 22 and 25. It can be observed that the variance of the baseline has been minimized and the spectral information has been clarified. Nevertheless, small differences appear, which can be attributed to the material and properties of the experimental setup. This has an impact on the accuracy of the models to be built.

The accuracy of the model depends on the quality of the data. The spectral variance of the same PG sample increases the probability of false classification. The reconstruction of mixed sample spectra may also fail due to faulty component spectra.

### 2.4. Simulation of Mix Spectra

To establish a machine-learning-based approach to classify different PG and determine the proportion of their mixtures, a sufficient amount of data is required for fitting. In order to save time and effort, simulated data are used to train models. We use a spectra simulator to generate suitable mixture spectra, taking the raw component spectra of each PG, as described in Section 2.3. In our examination, we include two different measurement settings to be simulated. The first assumes that the spectrometer observes a surface with a binary mixture of one PG and a metal surface. On the other hand, the spectrometer observes a surface with a binary mixture of only two PGs (second measurement setting). As in both cases we regard mixtures of two components, the metal surface is treated as an additional granulate. Thus, a total of 25 different components and the sheet metal are investigated.

In the following, we proceed with the calculation of the mixed spectra and summarize the entire process in the Algorithm 1. For simplicity, we restrict ourselves to present a calculation of one mixture spectrum of two components. The proposed method can subsequently be repeated for each mixture of interest with an arbitrary number of corresponding spectra samples.
**Algorithm 1:** Spectra mix  **Data**: **C**    set of two PGs to mix    **s**, **sr**    sample and reference spectra    **inttime**    list of individual integration times    **N**    number of samples to be simulated  **Result**: **sm**    spectra mix of binary combination **C**  (  $p_{c}\leftarrow get\_proportion(N)$         //( compute shares per component for every mixture  $a_{c}\leftarrow get\_inttime\_factors(\mathrm{inttime},p_{c})$         //( calculate integration time factors  $s_{c},{\mathrm{sr}}_{c}\leftarrow get\_spectra\_sample(s,\mathrm{sr},N)$         //( choose randomly component spectra  ${\mathrm{sm}}_{c}\leftarrow get\_spectra\_mix(s_{c},{\mathrm{sr}}_{c},a_{c},p_{c})$         //( compute mix spectra (

Considering *C* as the set of components *i* of one PGs mixture, the gained reflectance spectrum is given by $s_{i}$ and the teflon reference spectrum by $sr_{i}$. Each mixture spectrum $sm_{C}$ is simulated as a linear combination of the associated component spectra.

Due to the applied dynamic integration time optimizer used, we have to adapt each component spectrum by a specific integration time factor $a_{i}$ to modify the spectrum with respect to the integration time of the obtained mixture spectrum. We assume that the integration time of the mixed spectrum $t_{C}$ depends directly on the proportion $p_{i}$ of each component. Furthermore, we assume that the amount of the detected signal is a linear function of time. The factors $a_{i}$ are the ratio between the theoretically determined integration time of the mixed spectra and the measurement time $t_{i}$ of the considered component and given in Equation (3):

(3) $$a_{i}=\frac{t_{C}}{t_{i}}\;\forall i\in C,$$

where

(4) $$t_{C}=\sum_{i\in C}p_{i}*t_{i}$$

and restricted to $\sum_{i\in C}p_{i}=1$ and $0\leq p_{i}\geq 1,\;\forall i\in C$. Thus, if $t_{i}>t_{C}$, the adaption factor is higher than 1, which indicates that the spectrometer receives more signals, otherwise $a_{i}<1$.

In the next step, the computation of the mix spectrum is denoted by Equation (5):

(5) $$sm_{C}=\sum_{i\in C}\log[1/\left(a_{i}*s_{i}/sr_{i}\right)]*p_{i}.$$

For calculation, we operate with the transformation of reflectance into absorbance.

To simulate a sufficient number *N* of mixture spectra for each combination of PGs, we proceed as follows: we set the proportion of the first component $p_{1}$ by randomly choosing *N* samples from a uniform distribution, such that for each $p_{1}$ holds $p_{1}\overset{\mathrm{iid}}{∼}U\left(0,1\right)$. The share of the other component is then automatically given by $1-p_{1}$.

Since we have a reservoir of 50 raw spectra for each PG, we also randomly select the component spectrum employed in each iteration. This random selection ensures that we account for the measurement variance of the experimental setup in our analysis. In summary, we create a batch of 250 mixed spectra for each combination. To replicate the results, we use random seeds in the software.

**Figure 5:**
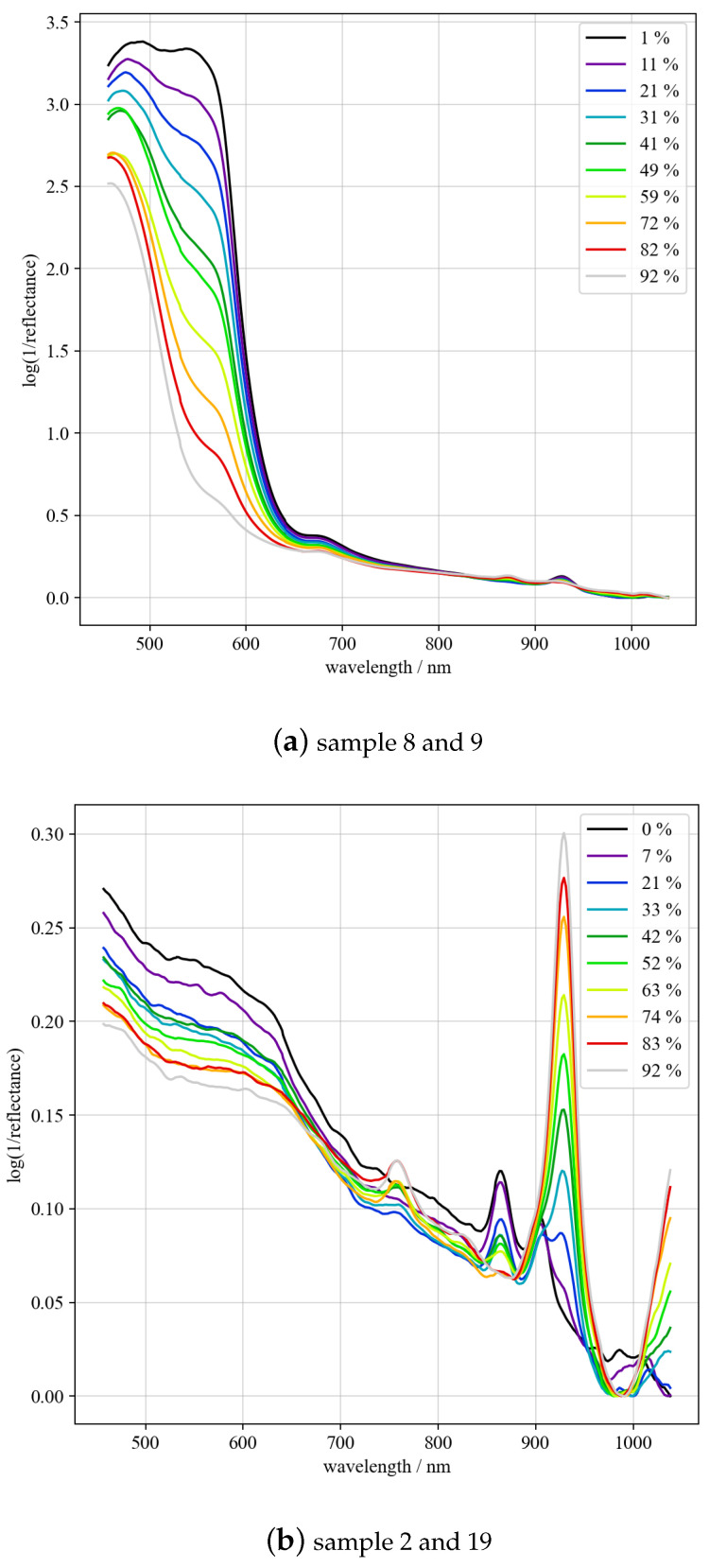
Simulated mix spectra of samples 8 and 9 (**a**) and 2 and 19 (**b**) with different component ratios.

For demonstration purposes, Figure 5 shows two sets of simulated mixed spectra based on samples 8 and 9 (a) as well as 2 and 19 (b), recorded by Vis29. Samples 8 (PS, opaque, cylindrical, and yellow) and 9 (PE, opaque, cylindrical, and red) are colored. Therefore, an increasing broad band at 550 nm is observed, which is due to the increase in 9 as compared to 8. Changes also occur in the NIR range due to the difference in absorption between PS and PE. Sample 2 (PE, transparent, cylindrical, and clear) and 19 (PC, opaque, cylindrical, and light gray) are not colored. As can be observed in the NIR range, the absorbance at 930 nm increases as the ratio of 19 to 2 rises.

### 2.5. Modeling

This subsection is dedicated to the algorithms and model architecture used in this study. As described in Section 2.4, we aim to identify the plastic mixtures and to detect the shares of the individual components.

For the classification, we employ a deep learning approach in the form of a one dimensional convolutional neural network (1D-CNN). For the determination of the proportions, partial least squares regression (PLS) is used. To validate the results, a reconstruction of the spectra is performed and subsequently presented.

#### 2.5.1. 1D-CNN

The application of 1D-CNN is common in the field of spectral data. The large majority of these studies is concerned with the exploration of soils and their properties (cf. among others, Kawamura et al. [24] and Wang et al. [25]). In recent years, the research has also increasingly focused on the classification of PG and waste, respectively [26,27,28].

In general, convolutional neural network (CNN)s are hierarchical architectures consisting of several hidden layers embedded by an input and output layer. In contrast to conventional artificial neural networks, the hidden layers of CNNs are a composition of convolutional layers, fully connected layers, and often pooling layers. For a thorough discussion, we refer to Malek [29] and Kiranyaz [30] for further information on this topic.

As the basis of the 1D-CNN architecture, we resort to the approach proposed by Ng et al. [31], because this architecture has already achieved promising results in the classification of plastics, elaborated by Neo et al. [32]. The 1D-CNN contains four convolutional layers, four max-pooling layers and two fully connected layers. To reduce overfitting, two dropout rates of 0.4 and 0.2 are additionally implemented. All hidden layers use a rectified linear unit (ReLU) as an activation function. The output layer uses a softmax function to generate the classification result.

The detailed description of the architecture is presented in Table 1.

For NIR data, the filter size of the pooling layers is set to two due to the smaller number of wavelengths of the Nir22 spectrometer. We use raw spectra as input data because CNNs generally show strong performance on unprocessed data, as outlined by Jernelv et al. [33]. Furthermore, this approach saves computational effort.

#### 2.5.2. PLS

The concept of a PLS is to predict a set of dependent variables *y* from a set of independent variables or predictors *X*. The prediction is generated by extracting a set of orthogonal factors, called latent variables, from the predictors. These latent components are specified to explain as much of the covariance between the features and the regression target [34] as possible.

In the proposed experiment, the collected spectra are the covariates *X*, and the proportions of one PG are the dependent variables *y* to be predicted. We do not apply multi-target regression because predicting the proportion of a component automatically determines the binary mixture (cf. Section 2.4).

In contrast to the classifier, we apply a Savitzky–Golay filter to smooth the spectra before training the model [23]. A property of this smoothing technique is that the filter can also be used to derive the data to remove both additive and multiplicative effects in the spectra [35].

The Savitzky–Golay filter requires the specification of certain smoothing parameters, such as the window length and degree of polynomial function used. To find the best combination of preprocessing and PLS parameters, we apply hyperparameter tuning by implementing a grid search algorithm. The underlying idea is to test all possible combinations of hyperparameters and use these candidates for fitting the estimator which maximizes the model accuracy that is assessed by an appropriate score. We allow 2 to 8 PLS components as possible parameters and none, first, and second derivatives for the Savitzky–Golay filter. We set the polyorder of 2 and window length of 3 for the filter as fixed parameters to keep the computational effort within manageable limits.

#### 2.5.3. Reconstruction of Spectra

1D-CNN and PLS will always provide a result even it is wrong. To check the results of both algorithms, the sample spectrum is reconstructed using the estimated components. linear least squares fitting (LSF) is used for the reconstruction. It is assumed that the mixed spectrum is a linear combination of two weighted component spectra identified by 1D-CNN. Each weight is calculated by minimizing the sum of the squared residuals of the sample spectrum and the sum of the weighted component spectra. The determined weights can be used to validate the PLS results. The sum of squared residuals is compared to an empirical threshold and used to validate the 1D-CNN result.

#### 2.5.4. Fusion Model

In a final step, the individual elements are combined to form a model. Each element is a separate step in the data stream. The design of this fusion model is a straight-forward data flow, which means that the individual segments are connected in a series.

Starting from a single unknown binary mix spectrum as the input data, the 1D-CNN classifies the components of the PG in a first processing step. After this information is obtained, the appropriate PLS regressor is selected and the proportions of the two identified components are estimated. Fitting an estimator for each possible binary mixture has the advantage of providing much more accurate prediction results vs. using one general regressor.

Since we assume that the 1D-CNN correctly performs the PGs, it is important to check this result, since the choice of the regressor is based on this decision. In other words, an incorrect classification result will also lead to an inaccurate prediction of the PGs proportions.

To reduce uncertainty and validate the results, we reconstruct the sample spectrum using the obtained PG types and proportions. For the reconstruction, we use the mean spectra over all collected sample spectra of a PG. This reconstructed mixed spectrum is afterwards compared with the input spectrum. If the the sum of squared residuals is less than a certain threshold, the model output is assumed to be true, meaning that the classified components correspond to the true labels. We refer to this part of the model as the validation element.

A further novel contribution of this model design is that a previously completely unknown sample can be easily and quickly detected as a foreign substance.

#### 2.5.5. Metrics

To assess the performance of models, we insert several quantitative metrics. These are well-known scores and are thus introduced briefly in the following.

In classification, we evaluate the estimator by computing the accuracy score (Equation (6)), which is defined as

(6) $$Accuracy\left(c,\hat{c}\right)=\frac{1}{N}\sum_{i=1}^{N}1(\hat{c_{i}}=c_{i}),$$

where 1(x) is the indicator function, $\hat{c_{i}}$ the predicted plastic mixture and $c_{i}$ the corresponding true class. The sum of correct predictions is divided by the total number of samples of *N*.

For the evaluation of the regression output, we establish multiple measures. To start with $R^{2}$, this score indicates the explained share of variance of the target variable *p* by the model. We constitute $p_{i}$ as the measured true proportion of PGs, and $\hat{p_{i}}$ is the predicted value. The metric is calculated over the full set of samples *N* and is given by Equation (7):

(7) $$R^{2}\left(p,\hat{p}\right)=1-\frac{\sum_{i=1}^{N}{\left(p_{i}-\hat{p_{i}}\right)}^{2}}{\sum_{i=1}^{N}{\left(p_{i}-\bar{p_{i}}\right)}^{2}},$$

where $\bar{p}=\frac{1}{N}\sum_{i=1}^{N}p_{i}$.

Furthermore, we compute the mean absolute error (MAE), which can be written as in Equation (8) below:

(8) $$MAE\left(p,\hat{p}\right)=\frac{1}{N}\sum_{i=1}^{N}\left|p_{i}-\hat{p_{i}}\right|.$$

As a final score, the mean squared error (MSE) is reported, as shown in Equation (9):

(9) $$MSE\left(p,\hat{p}\right)=\frac{1}{N}\sum_{i=1}^{N}{\left(p_{i}-\hat{p_{i}}\right)}^{2}.$$

#### 2.5.6. Software

The following software versions were used for data processing, model generation, and visualization: Python version 3.9.7 with packages of pandas 1.4.0, numpy 1.21.5, scikit learn 1.0.2, scipy 1.8.0, and matplotlib 3.5.1. We have designed our own scipy-based methods for the scikit learn pipeline API. To build the 1D-CNN, we resort to the Keras library, which is part of the TensorFlow 2.4.1 library. We use the Adam optimizer with a learning rate of 0.0002 for 250 epochs. The neural networks were trained using the categorical cross entropy as the loss function, which provides a one-hot representation of labels. For PLS, we operate with the scikit-learn package applying standard implementations. The validator uses the lsq_linear function from the scipy library for reconstruction.

All computations are carried out on a Intel Xeon Gold 6140 processor with 2.3 GHz and 8 virtual CPUs.

## 3. Results

The presentation of the results is split into the three different sections that together form the fusion model. In each subsection, we consider the model uncoupled from the data pipeline and evaluate its performance. Afterwards, we regard the ensemble of the different elements as a whole.

### 3.1. 1D-CNN as Classifier

As described in Section 2.4, we examine two special cases of binary mixtures. The first research environment is limited to mixtures between one PG and the metal surface as a background/sample holder. In the other, more complex setting, the focus is on binary mixtures of PGs-300 combinations in total.

To train and assess the model accuracy, we use 200 generated mixture spectra per combination for fitting the classifier1 and calculate the accuracy from the remaining spectra. However, it should be noted that, strictly speaking, the test data are not completely independent of the training samples since they are from the same population. Since two spectrometers covering different wavelength ranges are used for data acquisition, a model is estimated for each spectral class—VIS, NIR and VIS-NIR as a combination of both.

First, the fraction of correct classification for the “granulate on metal background” experiment is in Table 2. The 1D-CNN has to distinguish between 25 classes, whereas each class is associated by an individual PG.

For each spectral class, the rate of correctly identified PGs is higher than 90%. Thus, the classification algorithm is observed. For VIS, the scores are 0.9680 and 0.9336 for the NIR spectral data and 0.9776 for VIS-NIR, which is the combination of both.

As additional information, Table 2 contains information about the mean proportion of PG when spectra were misclassified. The values indicate that the 1D-CNN fails particularly for low-concentrated mixtures, i.e., the spectrometer detects nearly just the metal surface and only barely the PG. The significantly higher mean value for NIR is caused by the misclassification of a 92% PG portion.

Based on these results, performance is evaluated by considering only samples where the share of the PG exceeds a certain threshold (cf. Table 2). These scores suggest that the metric increases with a rising threshold. For VIS-NIR, the 1D-CNN has an accuracy score of over 99% from a limit of 2% plastic in the mixture. Considering the threshold of 5%, the algorithm achieves a near perfect classification performance for VIS and VIS-NIR. Hence, a minimum value of the share of PG can be set, above which classifier1 practically does not misclassify.

To proceed with the second investigation, where two PGs are mixed and the varying components are determined, we list the results in the same way as for classifier1 (cf. Table 3). In this setting, there are 300 types of mixtures with varied composition. In spite of this being an 8 times larger number of classes than in experiment 1, the algorithm indicates an accuracy of about 93% for VIS-NIR in the scope of all samples. In terms of VIS and NIR, the correct classification rate is 86.95% and 90.72%.

We also inspect the increasing occurrence of misclassified instances at low proportions of one component. The value presented in Table 3 is given by the mean over the minimum percentage of both components. A drop of samples with a minimum share in one component less than the same thresholds, as in the previous examination, and it also significantly improves the performance. As from a cut-off value of 3%, the VIS and VIS-NIR gain a level of correct classification of about 0.95. Analogous to classifier1, the highest scores are obtained by combining VIS and NIR spectra into VIS-NIR.

Further analyses demonstrate that the model achieves an accuracy of above 0.99 with a sensitivity of 7% for the VIS-NIR. Exploration of the misclassified combinations of mixed spectra reveals no group with a noticeably higher error rate.

### 3.2. PLS as Regressor

After evaluating the classifiers, we proceed by listing the results obtained for PLS in Table 4. We present values for the commonly used metrics coefficient of determination ($R^{2}$), mean absolute error (MAE), and mean square error (MSE).

In our model design, the regression estimator is downstream of 1D-CNN. In other words, this model element exhibits a different set of information compared to the classifier, as the components of the detected PG mixture are already known. Hence, we generate models for each combination between PG and the metal surface and for every binary mixture of PGs among themselves.

The results reported in Table 4 are the mean performance measures across all estimators, split by experiment. The error values are referenced on the component percentages. Analogous to 1D-CNN, we use 200 mixture spectra to train the model and evaluate the performance using a validation data set of 50 spectra for each combination. To assess the accuracy of the training data, we operate with a 10-fold cross validation.

Most striking are the very high $R^{2}$ values in all cases investigated, suggesting a perfect fit between measured and predicted samples. These results are in broad agreement with previous studies applying PLS to plastic data [21]. By inspecting the standard deviation, we find relatively weak volatility in the results. In the following, we focus on the validation data.

Focusing on other metrics shows that the MAE is less than 0.80% in all cases. Therefore, the model achieves high preciseness in predicting the proportions of PGs.

The results of the ranking by spectrometer indicate contrary results compared to the classifier, as NIR performs better than VIS, whereas VIS-NIR performs best in all categories.

When the metrics are broken down by type of experiment, the values exhibit slightly higher accuracy considering binary mixtures than rather PGs on a metal surface. This result should be treated with caution because we calculate the mean over two different sample sizes, 25 and 300, and therefore statistical effects could also have some impact.

The inspection of the choice of hyperparameters denotes eight PLS components in almost all cases, while there exists no clear structure in the remaining parameters to be searched.

### 3.3. Fusion of Models

After the assessment of the 1D-CNN and PLS, we concentrate on the series connection of the individual elements. To overcome misclassifications, we activate the validator as an additional segment in the data flow. As we have already discussed the performance of the classifier and the regressor, we focus on the reconstruction of the spectra as a validation tool.

Since the results of the first experiments suggest a near-perfect classification, we limit the presentation of the results to binary mixtures of PG to demonstrate the benefit of a validator in an appropriate setting. We also continue with VIS spectra and the combination of both spectral ranges, as preliminary examinations demonstrate the best results for these data. We set the threshold from which the classification output is declared faulty to 0.40 for VIS and 0.80 in the other case. These values have been demonstrated to be the most appropriate in previous simulations.

For evaluation, we take 100 randomly selected spectra from the validation data set. In general, we observe correct classification in 92 cases for VIS and 93 cases for VIS-NIR. Before inspecting the validator´s decision, we illustrate possible scenarios faced by the validator.

**Figure 6:**
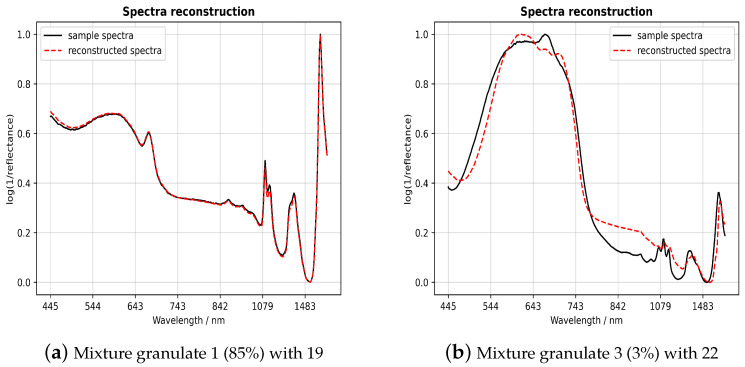
Reconstructed spectra of the established validator algorithm.

Figure 6a shows a normalized VIS-NIR spectrum of the mixed samples in 85% of 1 (PET, opaque, ellipsoidal, and gray) and 15% of 19 (PC, opaque, cylindrical, and gray) as a solid black line for illustration. The red dashed line is the reconstruction of the sample spectrum. As can be observed, the reconstruction differs only minimally from the sample spectrum. The sum of squared residuals is small and below the set threshold. Therefore, the validator returns this classification result as true.

For comparison, Figure 6b shows the opposite case. The reconstruction differs significantly from the sample spectrum, and the sum of the squared residuals is above the threshold. The result of the classifier is validated as false. It is worth mentioning that the proportion of sample 3 (PBT, opaque, ellipsoidal, and beige) is only 3%. Sample 22 (PC, opaque, cylindrical, and blue) dominates the sample spectrum with 97% and makes the correct determination of the minor component difficult. This is because of low SNR and a less specific spectrum, resulting in misclassification.

In general, at a constant integration time, a decreasing proportion of a component spectrum will lead to a low signal to noise ratio of that component. In addition, a second effect leads to a decrease in SNR. Due to the mixing of weighted component spectra, the SNR of a component that requires a longer integration time for the best SNR, will suffer from a short integration time. In cases where the integration times of the pure components are almost equal, the classification accuracy depends on the variance of the measurement.

**Figure 7:**
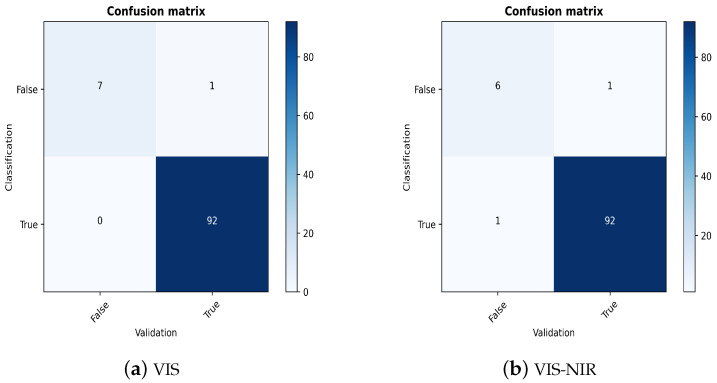
Confusion matrix of the validation process of binary plastic granulate mixtures for the two spectral classes.

As discussed previously, the validator generally helps to discover faulty classification results. We proceed by plotting the confusion matrices of the two spectral ranges investigated. Figure 7 reports values for a recall of 0.88 for VIS and 0.86 for VIS-NIR, which means that the validator is capable of detecting the wrong classification results with a very high percentage. In both cases, one spectrum is wrongly identified as correctly classified, although the predicted label does not correspond with the ground truth.

For precision, we observe 100% for the VIS application and 86% for VIS-NIR, which also implies a high power to indicate accurately misclassified spectra (cf. Figure 7).

## 4. Discussion

In the first experiment, we start by distinguishing samples detected on a metal surface with different coverage rates. Then, we continue by considering binary mixtures of PG with different shares of each components.

To acquire a sufficient amount of data, we devise a spectra mixer to simulate a sufficient number of appropriate semi-empirical samples to fit the inserted models. The spectra simulator enables the generation of an arbitrary number of spectra consisting of multiple granules with varying shares of the components. We generated a total of 6125 and 75,000 semi-empiric mix spectra to train and validate a classifier and a regressor, respectively.

In a fusion model, we connect multiple estimators in a series in order to properly classify mixture and component proportions. For classification, we implement a 1D-CNN-based approach using a well documented and proven network architecture. To determine the proportions of PGs, we apply PLS. We evaluate the performance of single elements of this fusion model.

Starting with classification, we observe very high accuracy scores for both experiments. In the case of PG with a metal surface background, the accuracy achieves a performance of about 97%, and 93% for binary plastic mixtures. The ranking of the spectral data used indicates that the combination of both spectrometers gives the best performance, followed by VIS and NIR data. Moreover, the results indicate that the classification model seems to have some sensitivity to the share of one PG, from which the model acts nearly perfectly. The PLS is able to predict the percent components with very small error values.

To spot misclassified samples, we install an additional tool to verify the classification output. The concept of the validator is to reconstruct a sample spectrum based on the prediction results and compare the reconstructed spectrum with the shape of the ground truth spectra. Provided that the divergence, measured as the sum of squared residuals, is lower than a certain threshold, the results of the model are assumed to be true, which confirms that the classified PG corresponds to the true labels. We test the validator on VIS-NIR and VIS datasets in the experimental setting of plastic mixtures. The established validation method shows precision and recall values between 85% and 100%.

In summary, the idea of spectra reconstruction is a very convenient and helpful tool for identifying misclassified samples. Apart from assessing the classification result, the tool is an essential indicator for quickly finding foreign substances in the flow of starting materials.

The results of this work can be used to optimize the detection accuracy in several fields. This applies to single and multiple spectrometers as well as to hyperspectral imaging. This especially applies in cases where an observed sample spectrum results from closely spaced objects, such as overlapping plastic parts, label color, or thin label foil on plastic containers.

In spatial binning, the fusion model could help to correctly interpret mixed spectral data to increase the measurement speed.

## 5. Conclusions

In this paper, we present a new research approach for the detection and classification of PGs. Differently from previous studies on this topic, which investigated the discrimination of various pure plastic types, we tackle the research gap of plastic mixtures. More specifically, we examine binary mixtures of 25 different PGs in two experimental settings.

The establishment of a spectra simulator addresses one of the main problems in empirical studies, namely, having a sufficient number of samples. This is crucial in data preparation because accurate models can only be trained with a sufficient variety of data.

The use of VIS-NIR spectroscopy and the presented fusion model could be used as a concept for building analysers in general. In particular, extrusion machines would benefit from the application of this concept. As a quality assurance feature implemented in extrusion machines, it would help to avoid the loss of resources and production of plastic waste.

A major advantage of the fusion model is the ability to validate the classification results. A positive effect of the validator is its capability to detect unknown materials. It could then react in a predefined way. For example, it could issue a warning and stop production. However, it could also ask to learn a new material. Due to the simplicity of the semi-empirical training data for the fusion model, it is very easy and effortless to extend the model.

In the future, the proposed fusion model with its artificial intelligence elements could be tested and evaluated with real data generated from an industrial process.

## Figures and Tables

**Table 1:** Architecture of the one dimensional convolutional neural network used for classification.

Type	Filter Size	Number of Filters	Activation
Convolutional	10	32	ReLU
Max-Pooling	2	-	-
Convolutional	10	64	ReLU
Max-Pooling	3	-	-
Convolutional	10	128	ReLU
Max-Pooling	3	-	-
Convolutional	10	256	ReLU
Max-Pooling	3	-	-
Dropout (0.4)	-	-	-
Flatten	-	-	-
Fully connected	-	256	-
Dropout (0.2)	-	-	-
Fully connected	-	No. of classes	Softmax

**Table 2:** Classification results of plastic granulate on metal background for the three spectral classes VIS, NIR and VIS-NIR. In addition to the accuracy of experiment 1, the table also contains information about the mean proportion of component $p_{1}$, in the case of misclassification, as well as the conditional accuracy when $p_{1}$ is higher than a given threshold.

Spectra	Accuracy Score	Misclassification Mean Share $p_{1}$	Accuracy Score Share $p_{1}$ Higher than			
			1%	2%	3%	5%
**VIS**	0.9680	2.58%	0.9813	0.9861	0.9884	0.9941
**NIR**	0.9336	6.73%	0.9472	0.9557	0.9620	0.9747
**VIS-NIR**	0.9776	1.55%	0.9894	0.9934	0.9959	0.9992

**Table 3:** Classification results of classification of binary plastic granulate mixtures for the three spectral classes VIS, NIR and VIS-NIR. In addition to the accuracy of experiment 2, the table also contains information about the mean proportion of component $p_{1}$, in case of misclassification, as well as the conditional accuracy when $p_{1}$ is higher than a given threshold.

Spectra	Accuracy Score	Misclassification Mean Min. $p_{1}/p_{2}$	Accuracy Score Min. $p_{1}/p_{2}$ Higher than			
			1%	2%	3%	5%
**VIS**	0.9072	4.85%	0.9229	0.9375	0.9481	0.9645
**NIR**	0.8695	5.92%	0.8861	0.9043	0.9196	0.9429
**VIS-NIR**	0.9311	3.30%	0.9473	0.9618	0.9717	0.9843

**Table 4:** Results of the PLS regressor; regression of the proportions of binary plastic granulate mixtures for the two experimental settings; and the three spectral classes VIS, NIR, and VIS-NIR. The MAE and MSE denote deviations from percentage values.

	Metal Surface Background					
	$R_{\mathrm{cv}}^{2}$	${\mathrm{MAE}}_{\mathrm{cv}}$	${\mathrm{MSE}}_{\mathrm{cv}}$	$R_{\mathrm{val}}^{2}$	${\mathrm{MAE}}_{\mathrm{val}}$	${\mathrm{MSE}}_{\mathrm{val}}$
**VIS**	1.00	0.8069	1.4856	1.00	0.7677	1.2592
**NIR**	1.00	0.7465	1.0067	1.00	0.7458	1.0446
**VIS-NIR**	1.00	0.6604	0.8530	1.00	0.6183	0.7199
	**Binary Mixtures**					
**VIS**	1.00	0.6929	1.2769	1.00	0.6510	1.1244
**NIR**	1.00	0.6747	0.9165	1.00	0.6725	0.9075
**VIS-NIR**	1.00	0.5248	0.6395	1.00	0.5183	0.6597

## Data Availability

The data presented in this study are available on request from the corresponding author.
